# Turbulent Drag Reduction Using Anisotropic Permeable Substrates

**DOI:** 10.1007/s10494-018-9916-4

**Published:** 2018-05-17

**Authors:** G. Gómez-de-Segura, A. Sharma, R. García-Mayoral

**Affiliations:** Department of Engineering, University of Cambridge, Trumpigton St, Cambridge, CB2 1PZ UK

**Keywords:** Drag reduction, Permeable substrates, DNS, Kelvin-Helmholtz

## Abstract

The behaviour of turbulent flow over anisotropic permeable substrates is studied using linear stability analysis and direct numerical simulations (DNS). The flow within the permeable substrate is modelled using the Brinkman equation, which is solved analytically to obtain the boundary conditions at the substrate-channel interface for both the DNS and the stability analysis. The DNS results show that the drag-reducing effect of the permeable substrate, caused by preferential streamwise slip, can be offset by the wall-normal permeability of the substrate. The latter is associated with the presence of large spanwise structures, typically associated to a Kelvin-Helmholtz-like instability. Linear stability analysis is used as a predictive tool to capture the onset of these drag-increasing Kelvin-Helmholtz rollers. It is shown that the appearance of these rollers is essentially driven by the wall-normal permeability $K_{y}^{+}$. When realistic permeable substrates are considered, the transpiration at the substrate-channel interface is wavelength-dependent. For substrates with low $K_{y}^{+}$, the wavelength-dependent transpiration inhibits the formation of large spanwise structures at the characteristic scales of the Kelvin-Helmholtz-like instability, thereby reducing the negative impact of wall-normal permeability.

## Introduction

The development of complex surfaces for turbulent drag reduction has been an area of scientific interest in the last decades. Recently, anisotropic permeable substrates have been proposed for turbulent drag reduction by Abderrahaman-Elena and García-Mayoral [1]. They suggest that the drag reduction ability of porous substrates is governed by the mechanism established by Luchini et al. [2, 3] for riblets, and by Jiménez [4] for complex surfaces in general. Complex surfaces, such as permeable substrates, can produce non-zero velocities at the substrate-channel interface. For vanishingly small surface texture, the effect of the texture on the overlying flow can be represented by slip-boundary conditions at the interface,

1 $$\begin{array}{@{}rcl@{}} \left. u \right|_{y = 0} &=& \ell_{x} \left. \frac{\partial u}{\partial y} \right|_{y = 0} \end{array} $$

2 $$\begin{array}{@{}rcl@{}} \left. w \right|_{y = 0} &=& \ell_{z} \left. \frac{\partial w}{\partial y} \right|_{y = 0}, \end{array} $$

where *ℓ*_*x*_ and *ℓ*_*z*_ are the slip lengths in the streamwise and spanwise directions, that is, the depth below the interface where the tangential velocity becomes zero if linearly extrapolated from *y* = 0. From this, *ℓ*_*x*_ can be defined as the virtual origin for the streamwise mean flow, and *ℓ*_*z*_ as that for the quasi-streamwise vortices [3, 4].

If the overlying spanwise flow induced by quasi-streamwise vortices is hindered more than the streamwise flow (*ℓ*_*z*_ < *ℓ*_*x*_), the vortices are ‘pushed away’ from the virtual origin of the mean flow and the vortices are further away from the streamwise virtual origin compared to the conventional smooth wall case. This decreases the local momentum flux and thereby reduces friction drag, and is a common mechanism in drag-reducing surfaces such as riblets or superhydrophobic surfaces. Luchini et al. [3] and Jiménez [4] proposed that drag reduction (DR) is proportional to the offset between the streamwise and spanwise slip lengths,

3 $$ DR ={\Delta} c_{f}/c_{f} \approx \mu_{0} \left( \ell_{x}^{+} - \ell_{z}^{+} \right),  $$

where the superscript ‘ + ’ represents viscous units. For *Re*_*τ*_ ≈ 1000 − 10000, the constant of proportionality is *μ*_0_ ≈ 0.04. Note that changing the Reynolds number only affects the constant of proportionality in the above expression [5].

After an extensive study of the effect of streamwise and spanwise slips on wall friction, Fukagata et al. [6] and later Busse & Sandham [7] observed that the linear regime given by Eq. 3 is only valid up to slip lengths of the order of one wall unit. Beyond that, the effect of the spanwise slip begins to saturate. This effect can be accounted for by the use of an effective spanwise slip length in Eq. 3, $\ell _{z_{eff}}^{+} = \ell _{z}^{+}/(1 + \ell _{z}^{+}/4)$, as discussed in detail by Fairhall & García-Mayoral [8] in this same volume. The saturation is the reason why isotropic slip lengths beyond a few wall units can also decrease the drag, which is in agreement with the results reported by Min & Kim [9] and the DNSs presented in Section 4.

For the case of anisotropic substrates of small permeability, Abderrahaman-Elena and García-Mayoral [1] established a relationship between slip lengths and permeabilities. They concluded that for these type of surfaces, the drag-reduction expression from Eq. 3 yields

4 $$ DR \approx \mu_{0} \xi \left( \sqrt{K_{x}^{+}} - \sqrt{K_{z}^{+}} \right),  $$

where $K_{x}^{+}$ and $K_{z}^{+}$ are the streamwise and spanwise permeabilities measured in viscous units, and *ξ* is a property of the geometry of the porous medium which reflects the interconnectivity of the flow within the substrate. Abderrahaman-Elena and García-Mayoral [1] drew a distinction between ‘poorly connected’ and ‘highly connected’ media. The first would be made up of micro-ducts essentially not interconnected, so diffusive effects could not occur at scales larger than the pore size. On the other hand, highly connected media, such as a swarm of micro-obstacles, could have diffusive effects on scales larger than the pore size. For impermeable materials *ξ* = 0, while for highly connected materials *ξ* ≈ 1. The value of *ξ* stems from the models used to capture the effect of large-scale diffusion at the interface region of the permeable medium.

For poorly connected substrates, Darcy’s equation is adequate to model the flow within the substrate [10–12]. However, at the interface, Darcy’s model cannot capture the boundary layer formed by the flow within the porous medium and a ‘jump condition’ proposed by Beavers & Joseph [13] is then generally used. This ‘jump condition’ imposes a slip velocity at the porous-fluid interface, *U*_*slip*_, which is proportional to the external shear, $\left . dU/dy \right |_{y = 0^{+}} = \alpha _{BJ} / \sqrt {K_{x}} \left (U_{slip} - U_{Darcy} \right )$, where *U*_*Darcy*_ is Darcy’s velocity deep inside the porous coating and the empirically-determined parameter *α*_*BJ*_ accounts for the geometry of the porous material. In highly connected substrates, on the other hand, Brinkman’s equation provides a reasonable model for the flow within and at the interface of the permeable substrate [14, 15]. At the interface region, several studies have drawn equivalencies between the Beavers and Joseph’s ‘jump condition’ and Brinkman’s equation [1, 16, 17]. Taylor [17] and Neale and Nader [16] have shown that when the substrate is deep enough, Beavers &Joseph’s boundary condition can be obtained from Brinkman’s equation. It was shown by [1] that the parameter *ξ* from Eq. 4, which accounts for the microstructure of the porous substrate, is *ξ* = 1/*α*_*BJ*_ when Beavers & Joseph’s boundary condition is used or, equivalently, when Brinkman’s equation is used $\xi = \sqrt {\nu / \tilde {\nu }}$, where $\tilde {\nu }$ accounts for the effective macroscale viscosity of the fluid-solid matrix. According to Eq. 4, the design of porous coatings for the purpose of drag reduction would seek to maximise the streamwise permeability, $K_{x}^{+}$, and *ξ*, while maintaining a low spanwise permeability.

Other macroscopic approaches to model flow within a permeable substrate include the volume-averaged Navier-Stokes equations (VANS) [18] which were used in [19, 20] and homogenisation [21, 22]. In the current work however, we focus on highly connected permeable substrates, for which the Brinkman model is a simple but reasonable approximation. In addition, similar to [12, 16, 23], we assume $\tilde {\nu } \approx \nu $ or *ξ* ≈ 1, since such materials would have a better potential for drag reduction [1].

Both Darcy and Brinkman models are based on volume averaging, so they implicitly require that any small volume within the substrate includes a large number of pores. As the averaging volumes approach the interface with the free flow, this assumption would eventually break down. This problem is still controversial in the specialised literature [21, 22, 24], and often empirical jump conditions are used as discussed previously. For simplicity, here we will assume that pores are infinitely small, so the continuum hypothesis holds for any vanishingly small volume, and fluid variables are continuous across the interface. For this to hold, in practice we would require that the pores be vanishingly small compared to any near-wall turbulent lengthscale. In this limit, the flow within the substrate would be dominantly viscous. For larger pores, on the other hand, advective effects could not be neglected and would need to be included in the model, for instance through a Forchheimer term [25, 26]. In addition, when the lengthscales of near-wall turbulence and the substrate become comparable, the turbulent structures no longer feel the presence of the wall as a homogeneous slip and Eq. 4 begins to fail. For a given porous material, the permeability lengthscales in viscous units, $\sqrt {K_{x}^{+}}$ and $\sqrt {K_{z}^{+}}$, would increase as the Reynolds number increases, and they would eventually become comparable to the lengthscales of near-wall turbulence. Equation 4 would then cease to hold.

Both, the linear theory of [3] and [4], and the simulations of [6, 7] and [9], neglect the effect of wall-normal permeability. However, most real surfaces that create slip, such as riblets [27], porous substrates [28] or superhydrophobic surfaces [29], produce a non-zero wall-normal velocity. When considering a non-zero wall-normal velocity, the performance improvement for increasing $K_{x}^{+}$ would break down for a certain permeability as additional degrading mechanisms set in.

Several authors have noted the appearance of spanwise coherent rollers over permeable substrates [19, 30, 31]. Abderrahaman-Elena and García-Mayoral [1] proposed that the formation of these rollers, originating from a Kelvin-Helmholtz-like instability, would be a possible drag-degrading mechanism over anisotropic permeable substrates. They proposed a model to bound the maximum achievable drag reduction based on the appearance of this instability. Jiménez et al. [32] observed the formation of these rollers over substrates which were permeable only in the wall-normal direction, with an impedance *β* between the transpiration and the pressure,

5 $$ v = \beta p,  $$

inferring that the relaxation of the impermeability condition at the wall is sufficient to elicit the rollers. They also noted that the appearance of these rollers was associated with a substantial increase in drag at the surface. Similarly, [27] attributed the formation of Kelvin-Helmholtz-like instabilities over riblets to having non zero wall-normal velocity at the interface. They also identified this instability as the cause for breakdown of drag reduction over riblets. This phenomenon could explain the similarities in the drag reduction curves of riblets and seal fur [33], which can be viewed as an anisotropic permeable substrate preferentially aligned in the streamwise direction. Following [1], guidelines for optimal configurations of drag-reducing substrates, as well as rough estimates to bound the maximum drag reduction achievable can be obtained based on the threshold for the appearance of the Kelvin-Helmholtz-like instabilities.

In the current work, we aim to establish estimates of substrate permeabilities at which Kelvin-Helmholtz-like instabilities can be predicted to occur over highly connected, anisotropic permeable substrates, using linear stability analysis. Based on this analysis, a model to predict drag reduction is proposed. We also present results from preliminary DNSs of flow over such media. The paper is organised as follows. In Sections 2 and 3 we revisit the stability analysis performed by [1] and obtain new estimates for drag reduction based on the analysis for highly connected permeable substrates. Subsequently, we present the results from several DNSs in Section 4 to better understand the effect of permeable substrates on the overlying flow. The conclusions are summarised in Section 5.

## Flow Within the Porous Substrate

**Fig. 1:**
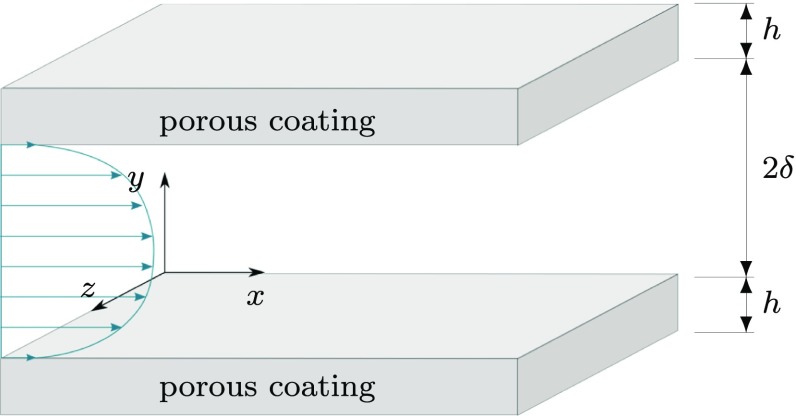
Geometry of the problem

We study channels of height 2*δ* delimited by two identical anisotropic permeable layers, characterised by their thickness *h* and their permeabilities *K*_*x*_,*K*_*y*_ and *K*_*z*_ in the streamwise *x*, wall-normal *y*, and spanwise *z* directions respectively, as portrayed in Fig. 1. We assume these to be the principal directions of the permeability tensor **K**. When **K** = 0 an impermeable medium is obtained, and when **K** →*∞* the medium offers no resistance to the flow.

The effect of a permeable substrate on the overlying flow can be represented by slip and impedance boundary conditions at the interface [1, 32, 34]. Here, we solve the flow within the permeable coating analytically to obtain the interface conditions that will be later implemented in the linear stability analysis and DNS of the channel.

We model the flow within the substrate using the Brinkman equation,

6 $$ \nabla p = - \nu \mathbf{K}^{-1} \mathbf{u} + \tilde{\nu} \nabla^{2} \mathbf{u},  $$

where **u** is the velocity vector, (*u*,*v*,*w*) being the velocity components in the streamwise *x*, wall-normal *y* and spanwise *z* directions, and *ν* and $\tilde {\nu }$ are the molecular viscosity of the fluid and the effective macroscale viscosity, respectively. The latter accounts for the viscosity of the fluid-solid matrix. Throughout this paper we will assume the density *ρ* to be unity for convenience.

The first two terms in Eq. 6 constitute Darcy’s equation, which results from a volume-average of the Stokes equation. It is a balance between the viscous drag due to the porous matrix and the pressure gradient across the porous medium, but it cannot capture the boundary layer that forms at the boundaries of the porous coating. The Brinkman term $\tilde {\nu } \nabla ^{2} \mathbf {u}$ accounts for diffusive effects on scales larger than those integrated through volume averaging into Darcy’s term, and allows for continuity of both the velocity and the shear stress at the boundaries. Also note that the Brinkman equation neglects any convective terms within the medium.

As depicted in Fig. 1, the porous layer is delimited by an impermeable wall on one end, where impermeability and no-slip conditions are applied, and by the interface with the channel on the other. At the substrate-channel interface, continuity of the tangential and normal stresses are applied. Therefore, for the substrate below *y* = 0, we have

7a $$\begin{array}{@{}rcl@{}} \nu \left[ \frac{\partial u}{\partial y} + \frac{\partial v}{\partial x} \right]_{y = 0^{+}} &=& \tilde{\nu} \left[ \frac{\partial u}{\partial y} + \frac{\partial v}{\partial x} \right]_{y = 0^{-}}, \end{array} $$

7b $$\begin{array}{@{}rcl@{}} \nu \left[ \frac{\partial w}{\partial y} + \frac{\partial v}{\partial z} \right]_{y = 0^{+}} &=& \tilde{\nu} \left[ \frac{\partial w}{\partial y} + \frac{\partial v}{\partial z} \right]_{y = 0^{-}}, \end{array} $$

7c $$\begin{array}{@{}rcl@{}} \left[ -p + 2 \nu \frac{\partial v}{\partial y} \right]_{y = 0^{+}} &=& \left[ -p + 2 \tilde{\nu} \frac{\partial v}{\partial y} \right]_{y = 0^{-}}, \end{array} $$

where positive and negative signs correspond to the channel and the porous sides of the interface, respectively. In addition, we also consider all the three velocity components to be continuous at the substrate-channel interface. The problem can be expanded using Fourier series in *x* and *z*, and assuming $\tilde {\nu } = \nu $, the continuity of tangential stresses reduces to that of $d\hat {u}/dy$ and $d\hat {w}/dy$, where the hat indicates variables in Fourier space, and the continuity of normal stresses to that of the pressure. Equation 6 can be solved by taking the divergence of the equation and combining it with continuity, together with the Brinkman equation in *y* and in *x*. From that a sixth order, bi-cubic equation for $\hat {v}$ can be obtained, which gives a general solution for $\hat {v}$ as a sum of exponentials. The general solutions for $\hat {u}, \hat {w}$ and $\hat {p}$ can be derived directly from that for $\hat {v}$. Considering the boundary conditions above, the analytical solution of the problem particularised at *y* = 0 provides the following relationships between the velocities and the pressure at the substrate-channel interface,

8a $$\begin{array}{@{}rcl@{}} \left. \hat{u} \right|_{y = 0^{+}} &=& \left. \hat{u} \right|_{y = 0^{-}} = \left. \mathcal{C}_{uu} \frac{d \hat{u}}{dy} \right|_{y = 0^{+}} + \left. \mathcal{C}_{uw} \frac{d \hat{w}}{dy} \right|_{y = 0^{+}} + \left. \mathcal{C}_{up} \hat{p}\right|_{y = 0^{+}}, \end{array} $$

8b $$\begin{array}{@{}rcl@{}} \left. \hat{w} \right|_{y = 0^{+}} &=& \left. \hat{w} \right|_{y = 0^{-}} = \left. \mathcal{C}_{wu} \frac{d \hat{u}}{dy} \right|_{y = 0^{+}} + \left. \mathcal{C}_{ww} \frac{d \hat{w}}{dy} \right|_{y = 0^{+}} + \left. \mathcal{C}_{wp} \hat{p}\right|_{y = 0^{+}}, \end{array} $$

8c $$\begin{array}{@{}rcl@{}} \left. \hat{v} \right|_{y = 0^{+}} &=& \left. \hat{v} \right|_{y = 0^{-}} = \left. \mathcal{C}_{vu} \frac{d \hat{u}}{dy} \right|_{y = 0^{+}} + \left. \mathcal{C}_{vw} \frac{d \hat{w}}{dy} \right|_{y = 0^{+}} + \left. \mathcal{C}_{vp} \hat{p}\right|_{y = 0^{+}}. \end{array} $$

The constants $\mathcal {C}_{ij}$ depend on the geometry of the permeable coating through *K*_*x*_,*K*_*y*_,*K*_*z*_ and *h*, but also on the streamwise and spanwise wavenumbers, *α*_*x*_ and *α*_*z*_. The corresponding expressions can be also obtained for the upper wall, at *y* = 2*δ*, by symmetry, and together they provide boundary conditions for the flow within the channel.

## Linear Stability Analysis

### Methodology

As mentioned before, the appearance of drag-increasing Kelvin Helmholtz rollers has been reported over permeable walls by several authors [19, 30, 32]. Therefore, in order to bound the range of optimum parameters, which will be investigated later in the DNSs, we aim to predict the formation of these spanwise-coherent rollers using linear stability analysis.

Following [27, 32] and [1], a linear stability analysis over a mean turbulent profile is performed to capture the formation of Kelvin-Helmholtz rollers. To approximate the mean turbulent profile, we use the analytic expression derived by Cess [35], which uses a molecular plus eddy viscosity *ν*_*T*_ = *ν*_*T*_(*y*) to match the typical turbulent profile of a channel. This profile and *ν*_*T*_(*y*) have been widely used in the literature [1, 36–38]. We present here a viscous stability analysis with viscosity *ν*_*T*_(*y*), but we have also used the molecular viscosity alone and similar results were obtained, with the only difference being the slightly larger value of the amplification reached for this case. It was shown in [1] that Squire’s theorem also holds when the boundary conditions are derived from a Darcy model for the substrate. The same cannot be formally deduced when including a Brinkman term, but based on previous evidence that the Kelvin-Helmholtz rollers are predominantly spanwise-coherent [1, 19, 20, 30, 32], we focus on spanwise-homogeneous modes and perform a two-dimensional, viscous analysis.

Linearising Navier-Stokes’ equations about the mean turbulent Cess profile and considering normal-mode solutions of the form $v^{\prime } = \hat {v} (y) \exp (i(\alpha x - \omega t))$, Orr-Sommerfeld’s equation for a variable eddy viscosity, *ν*_*T*_(*y*), is obtained,

9 $$\begin{array}{@{}rcl@{}} &&\left[\left( \alpha U - \omega \right) \left( D^{2} - \alpha^{2} \right) - \alpha \frac{d^{2} U}{dy^{2}} + i \nu_{T} \left( D^{2} - \alpha^{2} \right)^{2}\right.\\ && \left. + i 2 \frac{d \nu_{T}}{d y} \left( D^{3} - \alpha^{2} D \right) + i \frac{d^{2} \nu_{T}}{d y^{2}} \left( D^{2} + \alpha^{2} \right) \right] \hat{v} = 0, \end{array} $$

where *D* denotes *d*/*dy*. We consider a temporal linear stability analysis where the wavenumber *α*, or alternatively the wavelength *λ* = 2*π*/*α*, is real and the pulsation *ω* = *ω*_*r*_ + *iω*_*i*_ is complex. A perturbation is therefore unstable when *ω*_*i*_ is positive. At the boundaries, we impose continuity of the wall-normal and streamwise velocities. Note that the latter condition was not required in the inviscid analysis by Abderrahaman-Elena and García-Mayoral [1]. The corresponding velocities at the interface are derived from the flow within the permeable substrate in Section 2. For the lower wall of the channel, *y* = 0, the boundary conditions are given by particularising Eq. 8 for *w* = 0.

To solve Eq. 9, the wall-normal direction is discretised using Chebyshev polynomials. The analysis has been conducted at three different Reynolds numbers, *Re*_*τ*_ = *u*_*τ*_*δ*/*ν* = 180, 550 and 1000. In agreement with [27] and [1], the results obtained are found to be independent of Reynolds number when scaled in viscous units. Hereafter, only results for *Re*_*τ*_ = 180 are shown, using 256 Chebyshev collocation points.

The consideration of viscous effects modifies the problem qualitatively compared to the inviscid analysis in [1]. In that study, diffusive terms were not retained within the permeable substrate, which would be representative of a substrate with negligible pore interconnectivity. The flow was assumed to be inviscid and could slip freely over the substrate, so no shear could be transmitted to the flow within, and Eq. 9 reduced to Rayleigh’s equation with boundary conditions of the form $\left . \hat {v} \right |_{y = 0^{+}} = \left . \hat {v} \right |_{y = 0^{-}} \equiv \left . \mathcal {C}_{Darcy} \hat {p}\right |_{y = 0^{+}}$, while the tangential velocity could slip freely. In the present study, the full set of boundary conditions from Eq. 8a are applied.

### Results and discussion

**Fig. 2:**
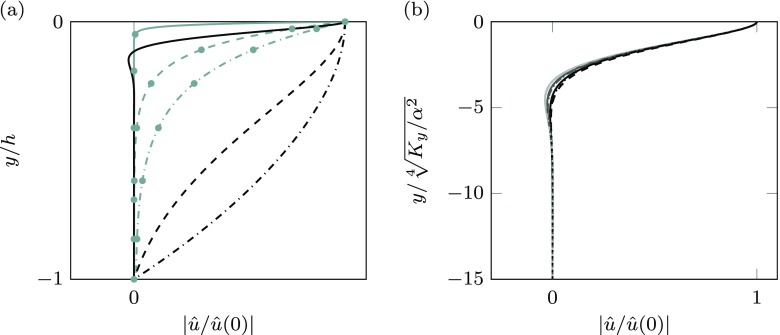
**a** Streamwise velocity profiles within the permeable substrate for $K_{x}^{+} = 100$ and $K_{y}^{+} = 1$, three different thicknesses: *h*^+^ = 5, – - –; *h*^+^ = 10, – –; *h*^+^ = 100,—. The wavenumber is *α*^+^ ≈ 0.1 (*λ*^+^ ≈ 60). Black lines, Brinkman’s solution; grey lines with circles, Darcy’s solution. **b** Self-similar streamwise velocity profiles within the permeable substrate for different coatings. Anisotropy ratios: $K_{x}^{+}/K_{y}^{+} = 20$, black line; $K_{x}^{+}/K_{y}^{+} = 10^{2}$, grey line; $K_{x}^{+}/K_{y}^{+} = 10^{3}$, light grey line. —, $K_{x}^{+}= 100$; – –, $K_{x}^{+}= 10$, for two different wavenumbers, *α*^+^ ≈ 0.1 and 0.2 (*λ*^+^ ≈ 30 and 60)

The main difference between using Darcy’s equation within the porous medium [1] and Brinkman’s equation results from the characterisation of the flow within the permeable substrate. To illustrate this, Fig. 2a portrays streamwise velocity profiles with and without Brinkman’s diffusive term, for substrates for which the solutions are particularly different. To emphasise the difference, values have been normalised with the maximum velocity, i.e. the velocity at the interface, $\hat {u}(0)$.

Darcy’s solution shows a large curvature, differing from the constant velocity profile typical of flows where Darcy’s equation is applicable. This discrepancy is due to the large variation of the streamwise pressure gradient, *∂p*/*∂x*, in the wall-normal direction, which is associated to $K_{y}^{+}$. Due to low wall-normal permeability, the pressure decays rapidly as it penetrates the coating. The streamwise pressure gradient is therefore weaker deep within the substrate than near the interface, and therefore drives less streamwise velocity, resulting in $\hat {u}$ profiles decaying rapidly with the depth, as shown in Fig. 2a. The effect is intensified for low wall-normal permeabilities, which are actually more efficient for drag reduction. For highly connected substrates with $\tilde {\nu } = \nu $, the Brinkman term $\tilde {\nu } \nabla ^{2} \mathbf {u}$ evaluated from these solutions would be non-negligible.

The flow within permeable substrates governed by the Brinkman equation is characterised by a ‘penetration depth’. This depth typically demarcates the region in which the diffusive terms are significant within the permeable substrates. This ‘penetration depth’ can be educed from the solution of the Brinkman equation. As mentioned above, the analytic solution of the Brinkman equation is a sum of exponentials, which splits into two regimes based on whether the anisotropy ratio, *K*_*x*_/*K*_*y*_, is greater or smaller than 1 + 1/(4*K*_*x*_*α*^2^). For the case of practical application for drag reduction discussed below, $K_{x} \alpha ^{2} \gtrsim 1 $ is satisfied, and the limit between the two regimes becomes roughly *K*_*x*_/*K*_*y*_ ≈ 1. Results for *K*_*x*_ ≪ *K*_*y*_ can be found in [39] and are qualitatively similar to those of [1]. When *K*_*x*_/*K*_*y*_ ≫ 1, the dominant term in the solution of the Brinkman equation is of the form $\hat {u}/\hat {u}(0) \propto exp(y/L_{p})$, where the penetration depth *L*_*p*_ is

10 $$ L_{p} = \sqrt{\frac{K_{x} \left( 2 \alpha^{2} + 1 + 2 \alpha^{2} K_{x} \sqrt{1 + 1/\left( K_{y} \alpha^{2} \right)} \right)}{ 4 \alpha^{2} K_{x} \left( K_{x}/K_{y} - 1 \right) -1}}.  $$

In the limit of permeable substrates of interest for drag reduction, *K*_*x*_ ≫ *K*_*y*_, we may also assume that (*K*_*y*_*α*^2^) ≪ 1 and $(K_{x} \alpha ^{2}) \gtrsim 1$. Within these constraints, Eq. 10 can be further simplified to $L_{p} \approx \sqrt [4]{K_{y}/(4\alpha ^{2})}$. Empirically, this parameter provides a good scaling for *L*_*p*_ in a wider range, even when $(K_{x} \alpha ^{2}) \gtrsim 1$ does not hold. The penetration depth is then essentially determined by the wall-normal permeability and the wavenumber. This scaling is demonstrated in Fig. 2b, where it collapses the normalised streamwise velocity profiles within the permeable substrate.

**Fig. 3:**
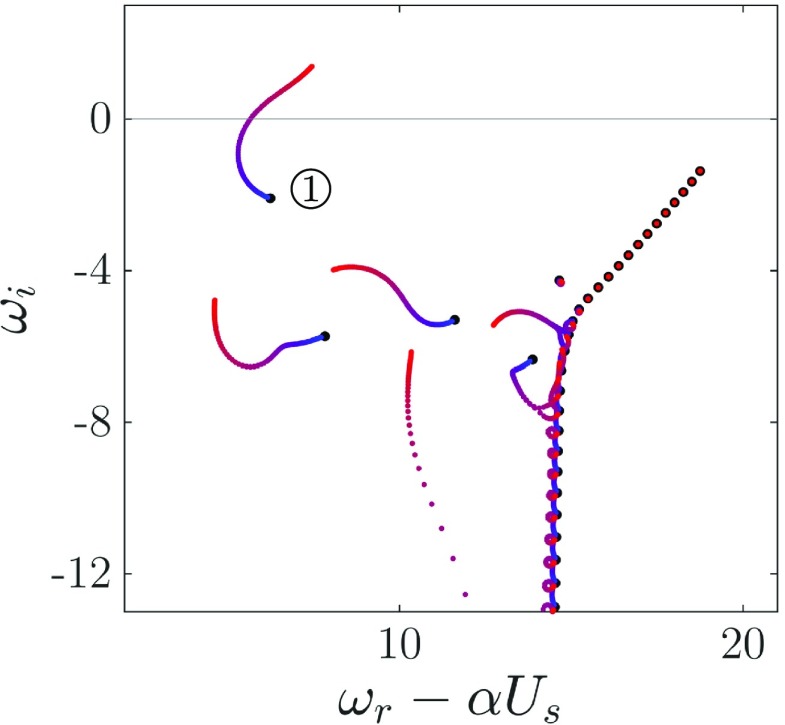
Trajectory of the eigenvalues - growth rate and frequency - for a given substrate with *K*_*x*_/*K*_*y*_ = 100 when varying $K_{x}^{+}$ and for *α*^+^ ≈ 0.1 (*λ*^+^ ≈ 60). Permeabilities increase from blue to red between $K_{x}^{+}= 0.01 - 10000$, and black points correspond to the smooth channel spectrum. Values are normalised using outer units, and *U*_*s*_ denotes the slip velocity at the substrate-channel interface

The presence of porous coatings at the limits of the channel alters the stable nature of the conventional turbulent smooth channel with impermeable walls. This can be observed in the results of the stability analysis. As both permeabilities $K_{x}^{+}$ and $K_{y}^{+}$ increase, the trajectories of the eigenvalues are portrayed in Fig. 3. When permeabilities tend to zero, the spectrum for an impermeable turbulent channel is recovered and it is found to be stable, in agreement with [40]. When increasing the permeabilities, however, there is one wall mode that becomes unstable, here labelled . On closer examination, we find that this eigenvalue is not just one mode, but rather that it consists of two similar modes, in agreement with what was observed by Jiménez et al. [32] –a varicose mode, symmetric with respect to the centreline of the channel, and a sinuous or antisymmetric mode. For small wavelengths both modes look the same. They start to diverge only at large wavelengths, due to the merging of the instability between the top and bottom walls of the channel. Jiménez et al. [32] argued that only the varicose mode would be equivalent to the one observed in turbulent boundary layers. Hence, in the present analysis we will focus on the varicose mode alone, although for the wavelengths of interest in the current work, *λ*^+^ ∼ 100, both modes are identical.

**Fig. 4:**
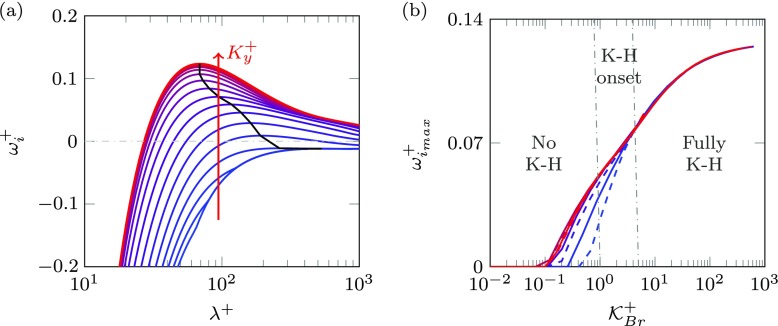
**a** Growth rate as a function of *λ*^+^ for *h*^+^ = 100 and $K_{x}^{+} = 100$. From blue to red the wall-normal permeability varies between $K_{y}^{+} = 0.01-5000$. **b** Maximum growth rate $\omega _{i_{max}}^{+}$ as a function of $\mathcal {K}_{Br}^{+}$ for different coatings. —, *h*^+^ = 100;– –, *h*^+^ = 10. From blue to red *K*_*x*_/*K*_*y*_ = 1, 10, 100 and 1000

**Fig. 5:**
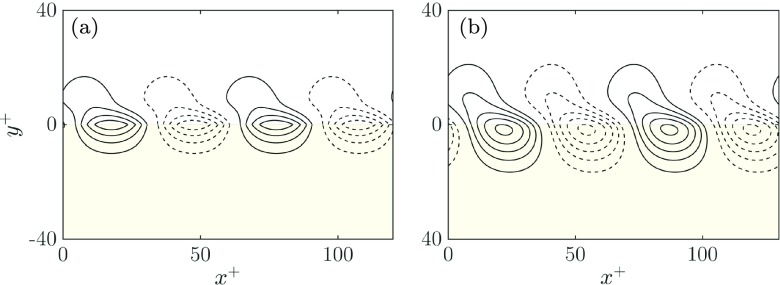
Equispaced isocontours of the streamfunction for the most amplified eigenvalue at *λ*^+^ = 68 and for a specific coating with $K_{x}^{+}= 100, K_{y}^{+}= 10$ and *h*^+^ = 100. Solid lines correspond to clockwise rotation, while dotted lines correspond to counterclockwise rotation. **a** Inviscid analysis. **b** Viscous analysis

Focusing on mode , Fig. 4a illustrates the growth rate $\omega _{i}^{+}$ as a function of the streamwise wavelength *λ*^+^ for different substrate configurations. The amplification increases as permeability increases, peaking at *λ*^+^ ≈ 70. In order to visualise this instability, the perturbation streamlines for the most amplified mode, at *λ*^+^ ≈ 70, are depicted in Fig. 5b. This mode consists of alternating rollers rotating clockwise and counter-clockwise separated by *λ*^+^ ≈ 70, which resemble Kelvin-Helmholtz-like rollers. The asymptotic value of the wavelength at which the maximum growth rate occurs corresponds to the wavelength of fully-developed Kelvin-Helmholtz rollers [1, 27]. This wavelength is set by the shear-length, which in turn is set by the shape of the mean velocity profile alone. The wavelength scales with the height at which the vorticity gradient, d^2^*U*/d*y*^2^, concentrates, $y_{c}^{+} \simeq 9$, in agreement with previous studies by [1, 27]. This value is independent of the Reynolds number, and is thus responsible for the scaling of the instability in viscous units.

In what follows we focus on the most unstable mode, as it is the dominant one. In Fig. 4a the most unstable mode for each coating is linked by the black line. To characterise permeable substrates easily, following [1] we propose a single, empirically fitted parameter to capture the influence of the substrate topology on the amplification of the most unstable mode,

11 $$ \mathcal{K}_{Br}^{+} = K_{y}^{+} \tanh \left( \frac{\sqrt{2 K_{x}^{+}}}{y_{c}^{+}} \right) \tanh^{2} \left( \frac{h^{+}}{\sqrt{12 K_{y}^{+}}} \right).  $$

Different coatings with the same value of $\mathcal {K}_{Br}^{+}$ exhibit essentially the same stability properties. Figure 4b portrays the amplification of the most unstable mode for a wide variety of coatings with different geometrical parameters *K*_*x*_,*K*_*y*_ and *h*. It shows that the effect of these parameters scales in viscous units and is essentially captured by $\mathcal {K}_{Br}^{+}$. In this figure, two different regimes can be distinguished, a low-permeability regime, $\mathcal {K}_{Br}^{+} \lesssim 0.2$, where the Kelvin-Helmholtz modes are not amplified, and a high-permeability regime, $\mathcal {K}_{Br}^{+} \gtrsim 100$, where the amplification of the Kelvin-Helmholtz instability asymptotes, which we refer to as the fully-amplified regime. We can then define an intermediate regime for the onset of the rollers. Note that to mark the asymptotic trend of the model, we have included large values of permeabilities for which our assumptions do no longer hold. Nevertheless, the relevant range for the present work is the intermediate one. Here, we are interested in coatings with *K*_*x*_ > *K*_*y*_ and large *h*^+^. In this case, the hyperbolic tangent terms in Eq. 11 are ≈ 1, and *h*^+^ and $K_{x}^{+}$ have little effect on the onset of the instabilities. The driving parameter becomes then essentially

12 $$ \mathcal{K}_{Br}^{+} \approx K_{y}^{+}. $$

This corroborates the scaling of the velocity profiles in Fig. 2b, where it was found that the lengthscale for the penetration depth, when *K*_*x*_ > *K*_*y*_, is set by $K_{y}^{+}$ and the wavelength alone. It is worth noting that the proposed parameter, $\mathcal {K}_{Br}^{+}$, differs from that proposed in [1], which is valid when diffusive effects are negligible in the substrate, and depends both on $K_{x}^{+}$ and $K_{y}^{+}$.

Furthermore, Fig. 4b shows that the onset of the instability occurs for an intermediate permeability, $\left .\mathcal {K}_{Br}^{+} \right |_{lim} \approx K_{y}^{+} \approx 1-5$. Beyond this threshold the Kelvin-Helmholtz rollers would be fully amplified, and would be expected to develop in the turbulent flow, increasing drag, as in [27].

In the study conducted by Abderrahaman-Elena & García-Mayoral [1], they performed an inviscid analysis to capture the onset of the Kelvin-Helmholtz rollers. This procedure has been traditionally used to capture the Kelvin-Helmholtz instability, as it is an inviscid phenomenon. Consistent with this hypothesis, an order of magnitude analysis shows that in the overlying channel region viscous terms are negligible compared to the advective ones at the typical wavelength and height of Kelvin-Helmholtz rollers. However, the analysis also shows that diffusive terms within the coating are not negligible for $\tilde {\nu }/\nu \simeq 1$, and qualitatively change the results. The major effect in the stability analysis of substrate diffusion being significant is that the leading parameter driving the onset of the Kelvin-Helmholtz-like instability becomes $K_{y}^{+}$, instead of $\sqrt {K_{x}^{+} K_{y}^{+}}$ as observed in [1]. Hence, although the viscous terms are negligible in the channel, the diffusive terms in the permeable substrate must be retained for highly connected permeable substrates. For materials with low diffusivity, in turn, the Darcy model of [1] would be more suitable.

Although not shown here, we have also conducted the linear stability analysis dropping the viscous terms within the channel, but retaining the Brinkman term within the substrate. The results are broadly similar to the viscous case (see, for instance the streamlines in Fig. 5), and we also recover $\left .\mathcal {K}_{Br}^{+} \right |_{lim} \approx K_{y}^{+}$ as the leading parameter for the onset of the instability. The main difference between the viscous and inviscid analyses lies in the value of the amplification of the instability. Differences are mainly significant for short wavelengths, which are damped more efficiently by viscosity. The smallest wavelengths, which are stable in the viscous analysis, are neutral in the inviscid analysis. However, for the wavelengths relevant in this problem, *λ*^+^ ≈ 70, we obtain essentially the same results.

Based on the parameter $\mathcal {K}_{Br}^{+}$, three different regimes can be identified for the instability. For small values of $\mathcal {K}_{Br}^{+}$, the instability is weak and would not be expected to manifest in the flow. For intermediate values, the instability would set in. For large values, the instability becomes fully amplified. These regimes are marked in Fig. 4b. The leading order parameter in the fully amplified regime, $ \mathcal {K}_{Br}^{+} \approx K_{y}^{+}$, and its limiting values for the three regimes remains unchanged for the viscous and inviscid analyses. For the inviscid stability analysis, the boundary conditions at the substrate-channel interface reduce to just $\hat {v}= \mathcal {C}_{vp} \hat {p}$, the streamwise velocity being free to slip. In the viscous case, an examination of our results indicates that $\mathcal {C}_{vu}, \mathcal {C}_{up}$ and $\mathcal {C}_{uu}$ do not significantly affect the formation of rollers, and that $\mathcal {C}_{vp}$ is the dominant coefficient. The results suggest that the appearance of rollers is essentially governed by the transpiration, while the tangential slips have a residual effect. Nevertheless, it should be noted that the values of $\mathcal {C}_{vp}$ are qualitatively different from those of the impedance coefficient derived from the solution of Darcy’s equation in [1].

### Establishing a limiting DR

In this section, we establish an upper limit for the drag reduction by combining the results obtained from the stability analysis and the model for predicting drag reduction discussed in Section 1. The methodology followed is similar to that used in [1], where the flow within the substrate was characterised by using Darcy’s equation. Here, we reassess the results when diffusion within the substrate is relevant.

**Fig. 6:**
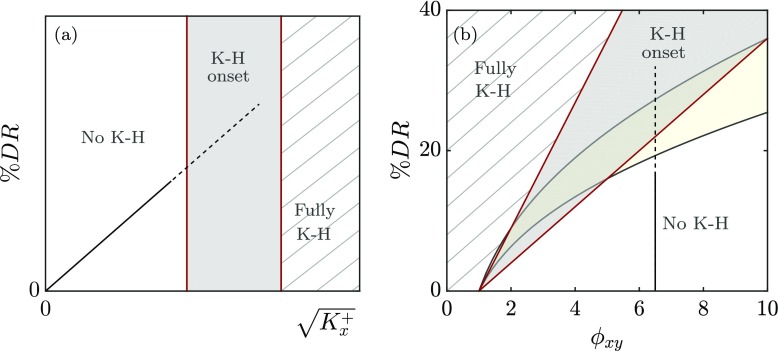
**a** Sketch of the predicted DR curve for a given anisotropy ratio $K_{x}^{+}/K_{z}^{+}$ as a function of $\sqrt {K_{x}^{+}}$. The straight line represents the linear behaviour defined by Eq. 4. The shading region corresponds to the values of $K_{x}^{+}$ for which Kelvin-Helmholtz (K-H) rollers are expected to set in. **b** Regions of no K-H, onset of K-H and fully amplified region with varying anisotropy ratios $\phi _{xy} = \sqrt {K_{x}^{+}/K_{y}^{+}}$. The shaded regions represent the limit for drag reduction set by the onset of Kevin-Helmholtz rollers. The grey region corresponds to the prediction using Brinkman’s equation, Eq. 13, and the yellow region that with Darcy’s [1]. The lower and upper bounds for the regions correspond to $ \mathcal {K}_{Br}^{+}= 1$ and 5, and $ \mathcal {K}_{Darcy}^{+}= 5$ and 10, respectively

Let us now consider an anisotropic permeable medium with a preferential permeability in *x*, and equal permeabilities in *y* and $z, K_{x}^{+} > K_{z}^{+} = K_{y}^{+}$, for sufficiently deep coatings, $h^{+} \gtrsim \sqrt {K_{x}^{+}}$, and highly connected porous media *ξ* ≈ 1. According to Eq. 4, for a given porous coating, i.e. with a fixed anisotropy ratio $\phi _{xy} = \sqrt {K_{x}^{+}/K_{y}^{+}}$, DR increases with $\sqrt {K_{x}^{+}}$, that is, as the Reynolds number increases for a frozen configuration. This behaviour is sketched in Fig. 6a, which would be analogous to classical drag reduction curves for riblets, such as those of [41], except that in those the degradation of DR for sufficiently large sizes could be clearly identified. With the present analysis, we can merely estimate the range of permeability lengthscales for which the degradation can be expected to occur, indicated in the figure with a shaded region, in the absence of earlier degrading phenomena. Assuming that up to the Kelvin-Helmholtz limit the performance given by Eq. 4 holds, we can estimate a tentative threshold for the maximum drag reduction. Substituting $\left .\mathcal {K}_{Br}^{+} \right |_{lim}$ for $K_{z}^{+}$ in Eq. 4, since $K_{z}^{+} = K_{y}^{+}$, yields

13 $$ DR_{lim} \leq 0.04\sqrt{\left.\mathcal{K}_{Br}^{+} \right|_{lim}} \left( \phi_{xy} -1 \right),  $$

which, once $\left .\mathcal {K}_{Br}^{+} \right |_{lim}$ is estimated, is a function of the anisotropy ratio $\phi _{xy} = \sqrt {K_{x}^{+}/K_{y}^{+}}$ alone, as depicted in Fig. 6b. In this figure, the shaded regions in grey and yellow represent the range of values for which the appearance of Kelvin-Helmholtz rollers would be expected for the present model and that of [1], respectively. For a fixed anisotropy ratio, following the drag reduction curve in Fig. 6a as $\sqrt {K_{x}^{+}}$ increases, would translate in Fig. 6b to moving vertically upwards, as sketched by the vertical line. The grey region would therefore correspond to the same shaded region as in Fig. 6a. We find that for highly connected porous substrates, the anisotropy effect is stronger than that predicted in [1] for coatings characterised by Darcy’s equation. For large anisotropy ratios, the current model produces less conservative bounds for *DR*_*lim*_. In the next section we will elaborate on the interaction of the permeable substrate with the near-wall flow using the results from preliminary DNSs.

## Preliminary DNS

In this section, results from preliminary DNSs are presented in order to investigate the interaction of porous substrates with the near-wall turbulence. Similar to the procedure followed in the stability analysis, we conduct simulations of channel flows with slip and transpiration boundary conditions. In particular, we evaluate the effect of a wall-normal transpiration combined with slip boundary conditions. We also focus on the differences between the wavelength-dependent boundary conditions obtained from resolving the flow within the substrates, and the homogeneous slip lengths and transpirations previously used in [7, 9] and [32].

### Numerical method

The DNS code is adapted from [27]. To solve the incompressible Navier-Stokes equations in a doubly-periodic channel of height 2*δ*, a fractional-step, Runge-Kutta method is used [42]. The spatial discretisation is spectral in *x* and *z*, and the wall-normal direction is discretised using a second-order centred finite difference scheme. Simulations are conducted at constant flow rate, starting from a smooth-wall flow at $Re_{\tau _{0}} \simeq 180$. The computational domain size is 2*π* × *π* × 2 in the streamwise, spanwise and wall-normal directions, respectively. A grid with 128 × 128 × 153 points is used, which corresponds to a resolution of ${\Delta } x_{0}^{+} \approx 8, {\Delta } z_{0}^{+} \approx 4$ and ${\Delta } y_{0}^{+} \simeq 0.3$ near the wall, while the wall-normal resolution increases to ${\Delta } y_{0}^{+} \simeq 3$ in the centre of the channel.

**Fig. 7:**
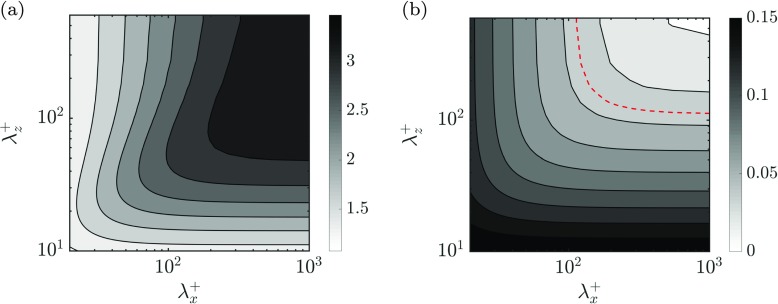
Boundary condition coefficients used for case ES as a function of $\lambda _{x}^{+}$ and $\lambda _{z}^{+}$ in viscous units. **a**$\mathcal {C}_{uu}^{+}$. **b**$-\mathcal {C}_{vp}^{+}$. The red dashed line in (**b**) is the contour where $-\mathcal {C}_{vp}^{+}$ is equal to the impedance coefficient from [32], used in the present work for case MK + J

The boundary conditions given by Eq. 8a are used to account for the presence of the permeable substrates. The coupling between the velocities and the pressure at the boundaries of the channel is implemented implicitly. Simulations are conducted for a constant mass flow rate in order to validate and compare the results with those obtained by Min & Kim [9] and Jiménez et al. [32]. The friction coefficient is defined as $C_{f} = 2 \tau _{w}/(\rho {U_{b}^{2}}) = 2 /(U_{b}^{+ 2})$, where the density, *ρ*, is chosen for convenience to be unity. The simulations are run for long enough so that *C*_*f*_ is obtained from averaging over statistically significant time windows, following the procedure described by Hoyas et al. [43]. The change in drag is given by the resulting change in the mean pressure gradient $\mathrm {d} \bar {p}/\mathrm {d} x$,

14 $$ {\Delta} D = \frac{C_{f} - C_{f_{0}}}{C_{f_{0}}} = \dfrac {\left( - \frac{\mathrm{d}\bar{p}}{\mathrm{d} x}\right) - \left( \left. - \frac{\mathrm{d}\bar{p}}{\mathrm{d} x} \right|_{0} \right)} {\left( \left. - \frac{\mathrm{d}\bar{p}}{\mathrm{d} x} \right|_{0}\right)},  $$

where the subscript 0 denotes the values for a smooth channel. In order to understand how the boundary conditions in Eq. 8a affect the near-wall flow, we consider the following three cases, for which the parameters are summarised in Table 1:

- MK, with homogeneous streamwise and spanwise slip lengths and zero transpiration, similar to one of the cases of Min & Kim [9], where the slips in both directions are equal, $\ell _{x}^{+} = \ell _{z}^{+} \approx 3.3$. This case allows us to validate the slip boundary conditions and later study the influence of the transpiration velocity separately from that of slip. The boundary conditions in the streamwise and spanwise directions are thus $u^{+} = \ell _{x}^{+} \partial u^{+}/\partial y$ and $w^{+} = \ell _{z}^{+} \partial w^{+}/\partial y$.
- MK + J, adds a homogeneous transpiration velocity to the previous case. The transpiration velocity is kept similar to one of the cases of Jiménez et al. [32], specifically, *v*^+^ = *β*^+^*p*^+^, where *β*^+^ = 0.043 is the impedance coefficient, homogeneous for all wavelengths. By comparing this case to MK, we evaluate the additional effect of a homogeneous transpiration.
- ES (Equivalent Substrate), with wavelength-dependent transpiration and slip lengths obtained from solving analytically the flow within the substrate, as modelled by Eq. 6. This yields the boundary conditions of Eq. 8a for the overlying flow. The depth and permeabilities of this substrate were selected to match the slip lengths of MK for the mean shear, in accordance with the linearised theory for drag reduction [1, 3, 32], and to match $\mathcal {C}_{vp}^{+}$ with the impedance coefficient of MK + J, *β*^+^, for the lengthscales for which the onset of Kelvin-Helmholtz rollers had previously been observed in DNS [27], $\lambda _{x}^{+} \approx 150$. This case corresponds to a porous substrate with $K_{x}^{+}=K_{z}^{+} \approx 13$ and $K_{y}^{+} \approx 0.1$. To illustrate how the slip and transpiration coefficients vary with the wall-parallel wavelengths, $\lambda _{x}^{+}$ and $\lambda _{z}^{+}$, maps of $\mathcal {C}_{uu}^{+}$ and $\mathcal {C}_{vp}^{+}$ are portrayed in Fig. 7. Since the values of the tangential permeabilities for case ES coincide, i.e. $K_{x}^{+}=K_{z}^{+}$, the equations for the flow within the substrate are invariant to a rotation in the *x* − *z* plane. $\mathcal {C}_{ww}^{+}$ is therefore equal to $\mathcal {C}_{uu}^{+}$ in Fig. 7a if $\lambda _{x}^{+}$ and $\lambda _{z}^{+}$ are interchanged.

**Table 1:** Coefficients for the boundary conditions for *u*^+^,*w*^+^ and *v*^+^ for the three cases studied, as defined in Eqs. 1, 2, 5 and 8

Case	*B* *C* *u*	*B* *C* *w*	*B* *C* *v*	*R* *e*_*τ*_	Δ*D%*
MK	$\ell _{x}^{+} = 3.3$	$\ell _{z}^{+} = 3.3$	0	165	− 15
MK + J	$\ell _{x}^{+} = 3.5$	$\ell _{z}^{+} = 3.5$	*β*^+^ = 0.043	184	+ 5
ES	$K_{x}^{+} = 11.3$	$K_{z}^{+} = 13$	$K_{y}^{+} = 0.1$	168	− 12

The Reynolds number *Re*_*τ*_, and the change in drag compared to a smooth channel at $Re_{\tau _{0}} \simeq 180$ are also included

In these preliminary simulations, for case ES, only the three coefficients from Eq. 8a which are assumed to drive the modifications in the flow physics, have been considered: $\mathcal {C}_{uu}^{+}$ and $\mathcal {C}_{ww}^{+}$, which represent the slip lengths in the streamwise and spanwise directions, respectively, as in [9] and [7], and the transpiration coefficient $\mathcal {C}_{vp}^{+}$, which relates the wall-normal velocity to the pressure fluctuations, as does the *β*^+^ coefficient in [32].

### Results and discussion

**Fig. 8:**
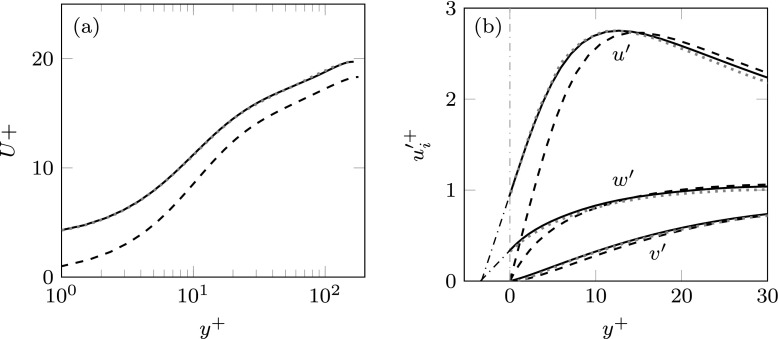
**a** Mean velocity profiles scaled with the corresponding *u*_*τ*_ at the substrate-channel interface. **b** rms velocity fluctuations scaled with the corresponding *u*_*τ*_. In (**a**) and (**b**) dashed line represents the smooth channel; dotted line, data from [9]; solid line, case MK

Throughout this section, viscous scaling is based on the shear velocity *u*_*τ*_, measured at the channel-substrate interface. Case MK replicates one of the simulations conducted by Min & Kim [9] for validation. The current results show good agreement with those of [9], as can be observed in the mean velocity profile and the rms velocity fluctuations depicted in Fig. 8. The figures also show results for a smooth channel for comparison.

**Fig. 9:**
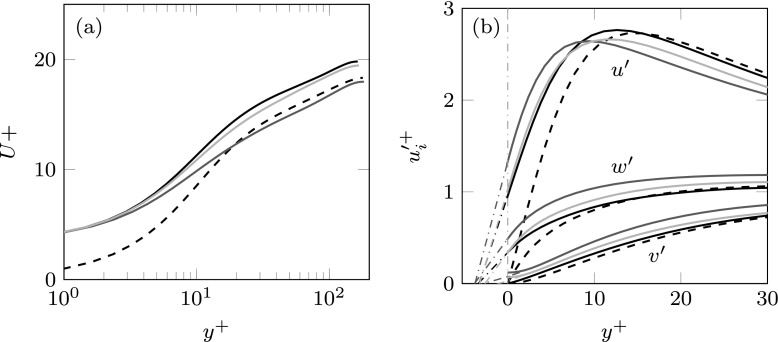
**a** Mean velocity profiles scaled with the corresponding *u*_*τ*_ at the substrate-channel interface. **b** rms velocity fluctuations scaled with the corresponding *u*_*τ*_. In (**a**) and (**b**) dashed line represents the smooth channel; black solid line, case MK; grey solid line, case MK + J; light grey solid line, case ES

The change in friction drag with respect to the smooth case is related to a shift of the intercept of the logarithmic velocity profile, Δ*D* ∝Δ*U*^+^, which according to the classical theory of wall turbulence is the only appreciable effect of the surface texture far away from the surface [44]. The change in friction drag for the cases under study can then be observed in the shift of the logarithmic velocity profile with respect to the smooth channel, as illustrated in Fig. 9a. An upward shift of the logarithmic region compared to the smooth channel, as for the cases MK and ES, results in a decrease of friction, and conversely for a downward shift, as for case MK + J. The shifts in the logarithmic region in Fig. 9a translate into the changes in drag reported in Table 1.

According to the linear theory, the reduction of drag is proportional to $\ell _{x}^{+} - \ell _{z}^{+}$ [3]. This theory is based on both slip lengths, $\ell _{x}^{+}$ and $\ell _{z}^{+}$, being vanishingly small. Note, however, that in case MK a net drag reduction is obtained in spite of both slip lengths being equal. This is due to the saturation effect of the spanwise slip beyond $\ell _{z}^{+} \simeq 1$, as mentioned in Section 1. Hence, for isotropic slip lengths of $\ell _{x}^{+} = \ell _{z}^{+} \approx 3$, as in case MK, the saturation of the spanwise slip leads to a decrease in drag. This effect is beyond the scope of the present work, but was thoroughly examined by Busse & Sandham [7].

On the other hand, under a wall-normal transpiration similar to that of [32], as in case MK + J, a slight increase in drag is observed, as the drag-decreasing effect of the slip lengths is outweighed by the drag-increasing effect of the wall-normal permeability. For case ES, wavelength-dependent interface conditions are obtained from the analytic solution of the Brinkman equation. The slips and transpiration are then different for different lengthscales. In particular, this case corresponds to a permeable substrate of $K_{x}^{+} = K_{z}^{+} \simeq 13$ and $K_{y}^{+} \simeq 0.1$. As previously stated, the value of the $K_{y}^{+}$ has been chosen to match $\mathcal {C}_{vp}^{+}$ with the transpiration coefficient of case MK + J for $\lambda _{x}^{+} \simeq 150$, i.e. for lengthscales for which the Kelvin-Helmholtz rollers have been previously observed [27]. The tangential permeabilities, $K_{x}^{+}$ and $K_{z}^{+}$, have been selected to match the slip lengths of the previous cases, MK and MK + J, for the mean shear. As observed in Fig. 9a, case ES recovers a significant drag decrease when compared to MK + J. The reason is that the transpiration coefficient, $\mathcal {C}_{vp}^{+}$, is smaller than the transpiration of case MK + J for the largest wavelengths, as shown in Fig. 7b, thereby inhibiting the formation of the drag increasing large-scale rollers observed in MK + J and [32].

The variations of the rms velocity fluctuations observed in Fig. 9b suggest a modification of the near-wall structures. The main effect of introducing slips and transpiration at the substrate-channel interface is a shift of the velocity profiles towards the wall. This shift can be quantified by extrapolating the profiles into the substrate as shown in the figure. The locations where the extrapolated velocity profiles go to zero correspond to the different component-wise virtual origins. Note also that for *u* and *w*, the virtual origins correspond to the streamwise and spanwise slip lengths, $\ell _{x}^{+}$ and $\ell _{z}^{+}$, respectively. While the virtual origins for *u* and *w* are approximately similar for the three cases, the origin of the wall-normal velocity differs. The greater the permeability, the deeper the virtual origin for *v* is. The origin of *v* is deepest in case MK + J, and consequently the positive effect of the slip lengths is more severely negated in this case. On the other hand, in Fig. 9b a change in the intensity of the fluctuations is also observed, mostly for the case with greater permeability – case MK + J. While for case MK the velocity profiles seem to be essentially those of the smooth channel shifted towards the wall by their corresponding virtual origins, the cases with greater wall-normal permeability, MK + J, and to a lesser extent ES, experience a modification in the intensities. The peak rms value of the streamwise velocity decreases, whereas *v*^′+^ and *w*^′+^ experience an increase, supporting the idea that the overlying flow is modified beyond a mere shift in the origin.

**Fig. 10:**
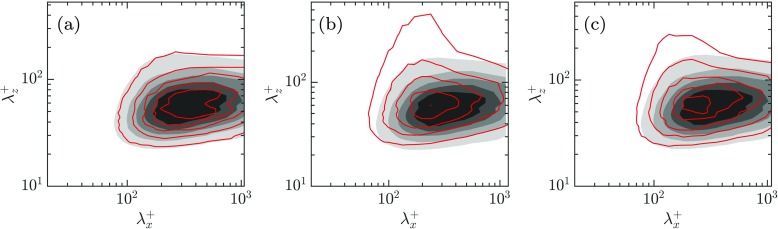
Premultiplied spectral density of the wall-normal velocity, *k*_*x*_*k*_*z*_*E*_*vv*_. Shaded, smooth channel at *y*^+^ = 7; lines, present DNSs shifted by the virtual origin of the wall-normal velocity. The contour increments in wall units are 0.00145. **a** MK at *y*^+^ = 6.6, **b** MK + J at *y*^+^ = 3.4 and **c** ES at *y*^+^ = 4.5

To gain further insight on the contribution to the rms of different lengthscales, a two-dimensional energy density spectrum of the vertical velocity near the wall is depicted in Fig. 10. As observed in Fig. 9b, the virtual origin of the wall-normal velocity differs from case to case. The relevant wall-normal location for comparison of the *v* spectra between the cases should be at the same height measured from the corresponding virtual origin of *v*. Therefore, the spectrum for the smooth channel at *y*^+^ ≈ 7, illustrated in greyscale, is superimposed over those for the present cases at $y^{+} = 7 - \ell _{v}^{+}$, where $\ell _{v}^{+}$ is the virtual origin of *v* fluctuations. The virtual origin $\ell _{v}^{+}$ for different cases can be observed in Fig. 9b and it is defined as the wall-normal displacement of the *v*^′+^ curve with respect to the smooth channel.

While case MK does not show a significant difference compared to the smooth channel, in case MK + J there is an accumulation of energy at $\lambda _{x}^{+} \approx 250$ and large $\lambda _{z}^{+}$. Although not shown here, this new energetic region decays at heights above *y*^+^ ≈ 25. The relaxation of the impermeability condition is responsible for the formation of spanwise coherent structures, and these in turn are responsible for the large increase of the spanwise and wall-normal fluctuations observed previously in Fig. 9b. For case ES, on the other hand, the energetic peak is slightly attenuated and displaced to lower wavelengths, $\lambda _{x}^{+} \approx 150$, similar to those observed over riblets in [27]. The weakening of the energetic region is due to the lower transpiration values imposed for large lengthscales by the wavelength-dependent transpiration coefficient, $\mathcal {C}_{vp}^{+}$, as shown in Fig. 7b.

**Fig. 11:**
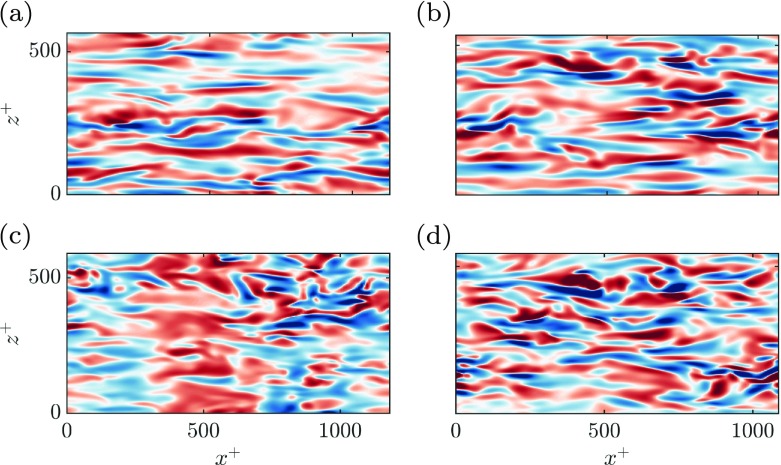
Instantaneous realisations of wall-normal velocity, *v*^+^, at a parallel plane to the wall. **a** Smooth channel at *y*^+^ = 7. **b** MK at *y*^+^ ≈ 6. **c** MK + J at *y*^+^ ≈ 3. **d** ES at *y*^+^ ≈ 5. In all cases, red to blue corresponds to [− 0.48,+ 0.48]

The presence of these energetic structures can also be shown in the instantaneous realisations of the wall-normal velocity depicted in Fig. 11. For case MK + J, there are elongated spanwise coherent structures separated by $\lambda _{x}^{+} \approx 250$. This alternating up-and-down motion can be associated to the Kelvin-Helmholtz instability studied in the first part of this paper and which was found to be governed by a single geometrical parameter – the wall-normal permeability $K_{y}^{+}$. Although not shown here, the strength and coherence of the streaky structures is disrupted by the new spanwise coherent structures, contributing to a drag degradation, as reported in [32], [27] and [30]. For case ES, however, the large rollers are inhibited due to a lower wall-normal permeability for the larger scales, and smaller spanwise structures appear instead. The smaller new energetic region observed in the spectrum in Fig. 10c compared to that in Fig. 10b suggests that their effect is not as significant. Although they are still detrimental for drag reduction, in Fig. 10 their impact seems to be smaller than in case MK + J.

## Conclusions

Following previous work by Abderrahaman-Elena & García-Mayoral [1], we have considered anisotropic permeable coatings for turbulent drag reduction. In the first part of the paper, we have modelled and analysed the development of drag-degrading Kelvin-Helmholtz instabilities over these substrates. The appearance of Kelvin-Helmholtz rollers is used to set an upper bound for the obtainable drag reduction. We have conducted a two-dimensional, fully viscous linear stability analysis of flow over permeable substrate. The flow within the substrate is characterised by the Brinkman equation. It is predicted that for coatings interesting for a drag reduction application, i.e. highly connected media preferentially permeable in the streamwise direction, the wall-normal permeability, $K_{y}^{+}$, is the single governing parameter for the triggering of Kelvin-Helmholtz-like rollers. The effect of anisotropy is stronger than predicted in [1] for substrates where diffusion is negligible, and higher bounds for the maximum achievable drag reduction are obtained.

Using the present analysis as a guideline for coating configurations, preliminary DNSs of channels with permeable substrates have been conducted. The DNSs confirm the formation of drag-increasing, spanwise-coherent structures due to the relaxation of wall-normal impermeability. When models based on substrates are considered, for which the transpiration is wavelength-dependent, the increase in drag is less than for a homogeneous transpiration. This is due to the lower transpiration for the larger structures, which decreases the turbulent mixing, and consequently also reduces drag.
